# Downregulating serine hydroxymethyltransferase 2 (SHMT2) suppresses tumorigenesis in human hepatocellular carcinoma

**DOI:** 10.18632/oncotarget.10415

**Published:** 2016-07-06

**Authors:** Chern Chiuh Woo, Way Cherng Chen, Xing Qi Teo, George K. Radda, Philip Teck Hock Lee

**Affiliations:** ^1^ Singapore Bioimaging Consortium, Singapore

**Keywords:** SHMT2, hepatocellular carcinoma, serine, glycine, 5,10-methylenetetrahydrofolate

## Abstract

Serine-glycine biosynthetic pathway diverts the glycolytic intermediate 3-phosphoglycerate to synthesize serine and glycine, of which the latter was found to correlate with cancer cell proliferation. Increased *de novo* biosynthesis of glycine by serine hydroxymethyltransferase 2 (SHMT2) is the central mechanism to fuel one-carbon pools supporting tumorigenesis. However, the therapeutic potential in targeting SHMT2 in hepatocellular carcinoma (HCC) is unknown. In this study we showed that SHMT2 inhibition significantly suppressed liver tumorigenesis. *In vitro*, SHMT2-knockdown was found to reduce cell growth and tumorigenicity in Huh-7 and HepG2 liver cancer cells. Moreover SHMT2-knockdown Huh-7 cells failed to form tumor xenograft after subcutaneous inoculation into nude mice. Similarly, inducible SHMT2 inhibition, via doxycycline-added drinking water, was found to reduce tumor incidence and tumor growth in a human tumor xenograft mouse model. SHMT2-knockdown increased the susceptibility of Huh-7 cells to doxorubicin suggesting its potential in combination chemotherapy. Through isotopomer tracing of [2–13C] glycine metabolism, we demonstrated that SHMT2 activity is associated with cancer phenotype. However, overexpression of SHMT2 was insufficient to transform immortalized hepatic cells to malignancy, suggesting that SHMT2 is one of the building blocks in liver cancer metabolism but does not initiate malignant transformation. Moreover, our results suggest that glycine, but not 5,10-methylenetetrahydrofolate, from the SHMT2-mediated enzymatic reaction is instrumental in tumorigenesis. Indeed, we found that SHMT2-knockdown cells exhibited increased glycine uptake. Taken together, our data suggest that SHMT2 may be a potential target in the treatment of human HCC.

## INTRODUCTION

Cumulative evidence suggests that cancer cells reprogram their metabolic pathways in order to sustain cell growth and proliferation [1, 2]. Increased glycolysis is a primary avenue to provide transformed cells with metabolic fuels that are needed to sustain biosynthetic pathways that generate precursors for macromolecular synthesis, such as the pentose phosphate pathway in ribonucleotide production, the regeneration of nicotinamide adenine dinucleotide (NAD^+^) from reduced nicotinamide adenine dinucleotide (NADH) through pyruvate reduction into lactate, and the serine-glycine biosynthetic pathway in one-carbon metabolism [3, 4]. Concomitant addiction to glutamine may also occur to support the elevated bioenergetics in cancer cells [5].

Deregulated cellular energetics is one of the emerging hallmarks of cancer as summarized by Hanahan and Weinberg [6]. In particular, the serine-glycine biosynthetic pathway plays a crucial role in DNA replication, and cancer cells are reprogrammed to hyper-activate this glycolytic shunt in driving oncogenesis. The phosphoglycerate dehydrogenase (PHGDH) gene, which catalyzes the oxidation of 3-phosphoglycerate to 3-phosphohydroxypyruvate [7], is amplified in a subset of human tumors including triple negative breast cancer and melanoma [8, 9]. Subsequent reactions are mediated by phosphoserine aminotransferase 1 (PSAT1) and phosphoserine phosphatase (PSPH) to yield serine [4]. Serine hydroxymethyltransferase (SHMT) next catalyzes the conversion of serine to glycine, simultaneously hydrolyzing tetrahydrofolate (THF) into 5,10-methylenetetrahydrofolate (MeTHF). Both SHMT1 and SHMT2 enzymes (cytosolic and mitochondrial isoforms respectively) are transcriptional targets of the oncogene c-Myc [10]. In particular, SHMT2 overexpression stimulates the proliferation of c-Myc deficient cells [10]. Conversely, its knockdown in Myc-dependent cells reduces NADPH/NADP^+^ ratio and results in a heightened level of reactive oxygen species, which in turn triggers hypoxia-induced cell death [11]. However, the effect of SHMT2 inhibition appears to depend on the cancer phenotype, in view of the suppression in cell proliferation of rapidly-proliferating LOX IMVI and HeLa cancer cells but not of the slowly-proliferating A498 cell line [12]. It has been proposed that the mechanism in SHMT2 knockdown involves the arrest of the Gl phase in cell cycle [12]. Loss of SHMT1 function on the contrary did not affect tumor development in mice with Apc^min^ and p53 knockout [13]. Moreover, SHMT1-deficient mice are viable and fertile, suggesting that it is not an essential source of THF-activated one-carbon units [14].

One of the key control reactions in cellular one-carbon metabolism is the decarboxylation of glycine and tetrahydrofolate (THF) into ammonia, carbon dioxide and 5,10-methylenetetrahydrofolate (MeTHF), catalyzed by glycine decarboxylase (GLDC). It has been shown that glycine metabolism strongly correlates with cancer cell proliferation [12]. Metabolic profiling data from 262 clinical samples indicated that sarcosine, which is an N-methyl derivative of glycine, is highly elevated in metastatic prostate cancer [15]. MeTHF plays an important role in folate metabolism which is tightly coupled to purine synthesis. Indeed, overexpression of GLDC in 3T3 mouse fibroblast cells resulted in cell transformation whereby these cells developed into tumors upon inoculation into nude mice [16]. Metabolic screening of 143 non-small cell lung cancer patient samples showed a positive correlation between GLDC expression and lung cancer mortality [16]. GLDC and SHMT1 positivity are associated with poorer prognosis in brain metastasis originated from breast cancer [17].

Examination of the gene expression of 1425 metabolic enzymes across NCI-60 cancer cell lines revealed that enzymes of glycine metabolism (including SHMT2, MTHFD2 and MTHFD1L) are highly expressed in rapidly-proliferating cancer cells. Since MeTHF is an important product of both GLDC and SHMT2 catalysis, its role in driving tumorigenesis warrants further investigation. In addition, the role of the serine-glycine biosynthetic pathway in liver cancer therapy is not known. In this study, we used a tetracycline-inducible SHMT2-knockdown tumor xenograft mouse model to demonstrate that inhibiting SHMT2 reduces tumor incidence as well as tumor growth. In addition we found that SHMT2 overexpression is insufficient to initiate malignant transformation and the tumorigenic potential of SHMT2 is mediated via glycine rather than MeTHF. Taken together, we propose that SHMT2 is a potential therapeutic target in the treatment of HCC.

## RESULTS

### Genes of serine-glycine biosynthetic pathway are upregulated in liver cancer cell lines

**Figure 1:**
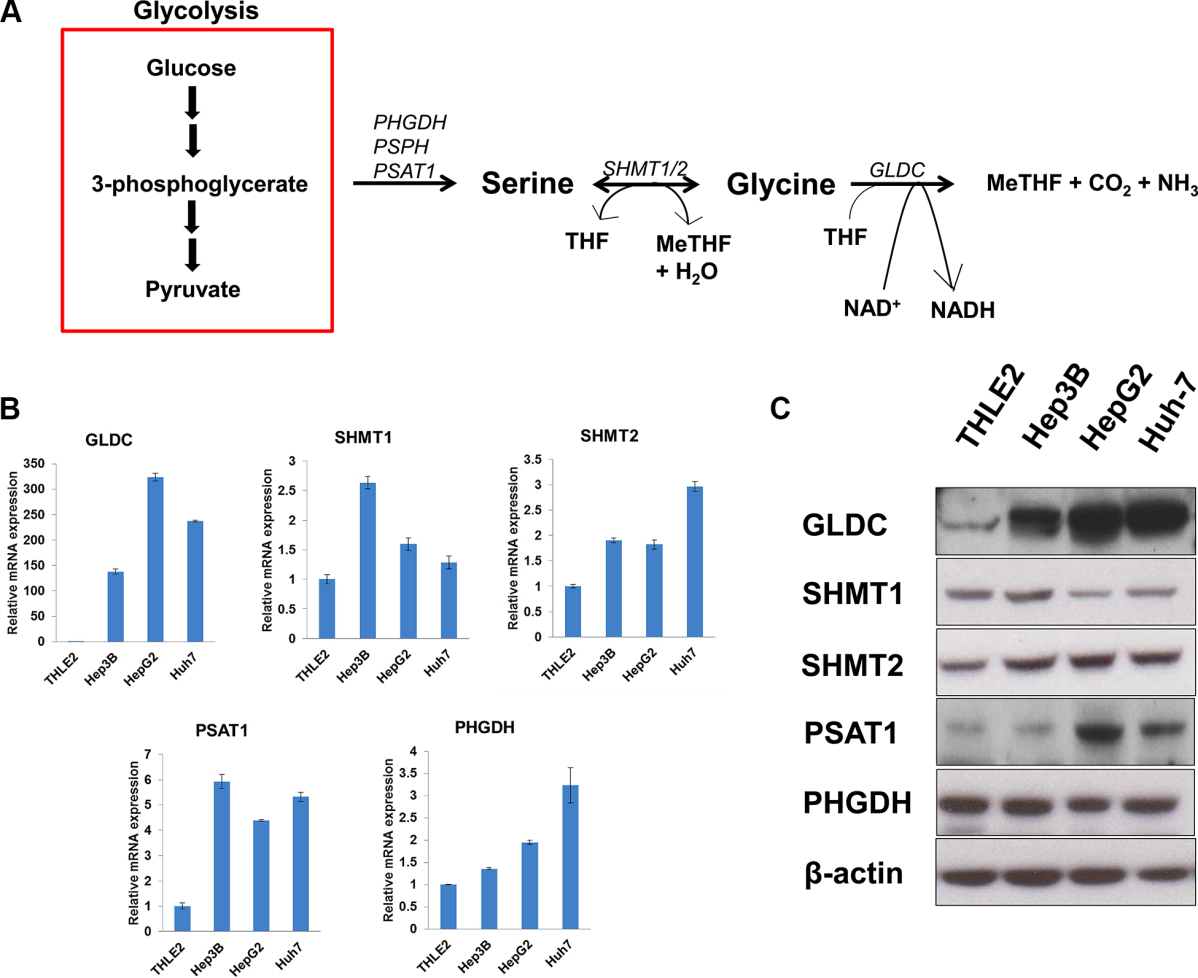
SHMT2 is upregulated in liver cancer cell lines (**A**) a brief outline of serine-glycine metabolism. (**B**) the mRNA expression of serine-glycine metabolic genes. The comparison was made between several liver cancer cell lines (Hep3B, HepG2 and Huh-7) in relative to an immortalized normal liver cell line (THLE2). The data represent mean ± SD of triplicate PCR reactions. (**C**) the protein expression of serine-glycine metabolic genes in several liver cancer cell lines versus THLE2 cells. The data are the best representative of three independent experiments.

The serine-glycine biosynthetic pathway starts by converting the glycolytic intermediate 3-phosphoglycerate into 3-phosphohydroxypyruvate via PHGDH, follow by conversion to serine and glycine as illustrated in Figure 1A. We first examined the gene and protein expressions of key metabolic enzymes of the serine-glycine biosynthetic pathway in three human HCC cell lines (Hep3B, HepG2 and Huh-7) and compared them to an immortalized normal liver cell line (THLE2). SHMT2 was found to be significantly upregulated at both mRNA and protein levels in the HCC cells (Figure 1B–1C), while the cytosolic isoform SHMT1 is upregulated in Hep3B cells only. GLDC is highly upregulated in all HCC cell lines in both mRNA and protein levels. This suggests the importance of GLDC in liver tumorigenesis, and this gene has been previously reported to positively correlate with lung cancer mortality [16]. While PSAT1 gene expression is increased in all HCC cell lines, the protein level is not elevated in Hep3B cells. HepG2 and Huh-7 cells showed significantly higher PHGDH mRNA level but no difference in protein expression was observed. Together, these data suggest that serine-glycine metabolic genes are upregulated to synthesize increased amount of serine and glycine to support liver tumorigenesis.

### Knockdown of SHMT2 suppresses cell growth and tumorigenicity in Huh-7 and HepG2 cells

**Figure 2:**
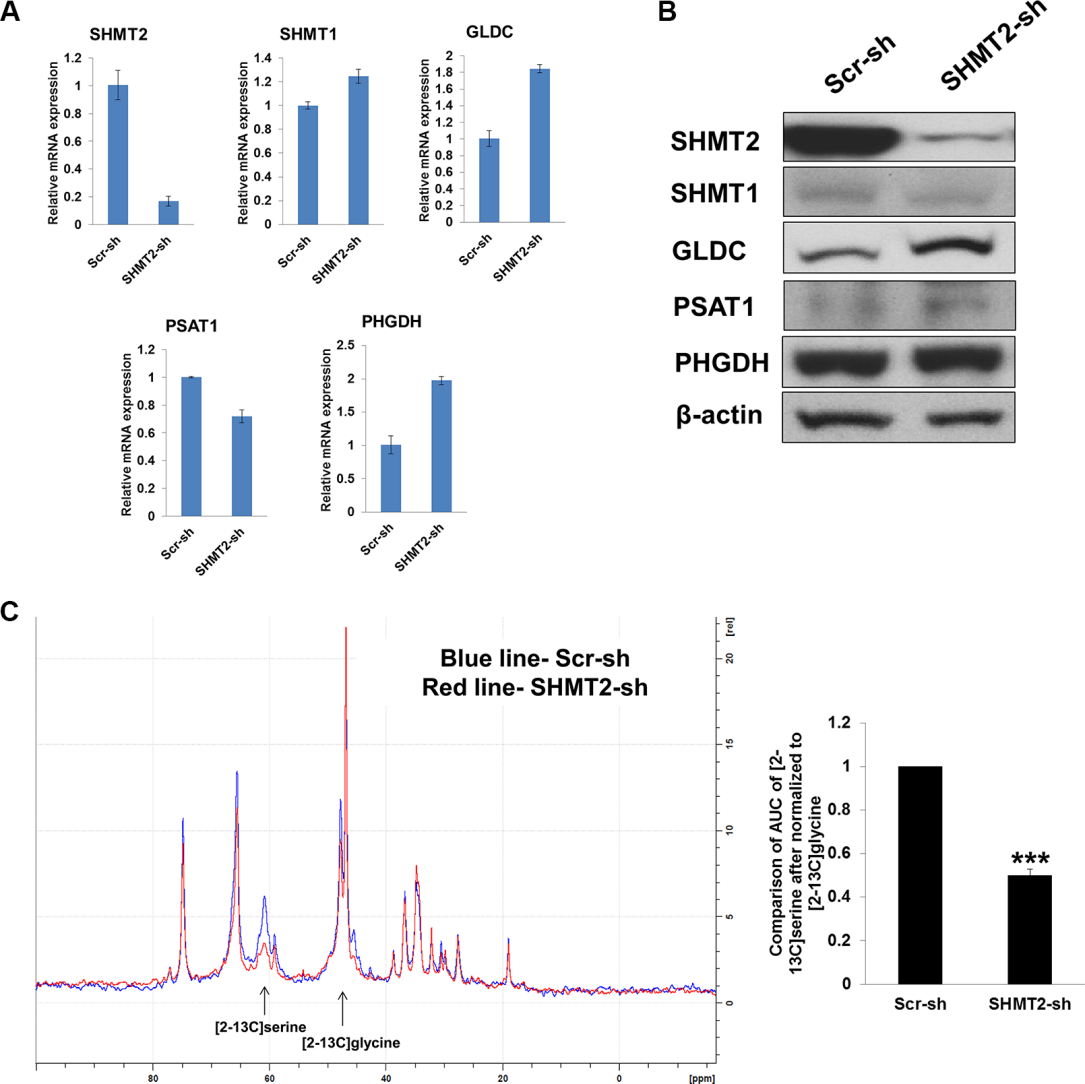
Effect of SHMT2 knockdown on serine-glycine metabolism in Huh-7 cells (**A**) the mRNA expression of serine-glycine metabolic genes in Huh-7 cells expressing SHMT2 shRNA (SHMT2-sh) in relative to Huh-7 cells expressing scramble shRNA (Scr-sh). The data represent mean ± SD of triplicate PCR reactions. (**B**) the protein expression of serine-glycine metabolic genes in Huh-7-SHMT2-sh cells versus Huh-7-Scr-sh cells. The data are the best representative of three independent experiments. (**C**) NMR spectrum (left panel) of Huh-7-SHMT2-sh cells versus Huh-7-Scr-sh cells. Bar graph (right panel) showing the relative comparison of AUC of [2–13C] serine (after normalized to [2–13C] glycine) of Huh-7-SHMT2-sh cells versus Huh-7-Scr-sh cells. ***p < 0.001.

We started by investigating the therapeutic effect of inhibiting SHMT2 in liver cancer cell proliferation and tumorigenicity. We successfully created stable SHMT2 knockdown in Huh-7 (Figure 2A–2B) and HepG2 cells (Supplementary Figure 1A–1B) with at least 80% reduction in mRNA and protein levels. We found that GLDC expression concomitantly increased in the SHMT2-knockdown cells. No change in SHMT1 expression suggests that the loss of SHMT2 could not be compensated by its cytosolic isoform. To study whether the activity of SHMT2 is indeed reduced after its knockdown, we incubated the cells with [2–13C] glycine because SHMT2 reversibly converts [2–13C] glycine to [2–13C] serine, which can be detected by C13-NMR spectroscopy. Our NMR data showed a significant reduction in [2–13C] serine level in SHMT2-knockdown cells as compared to the control cells indicating a decrease in SHMT2 activity (Figure 2C).

**Figure 3:**
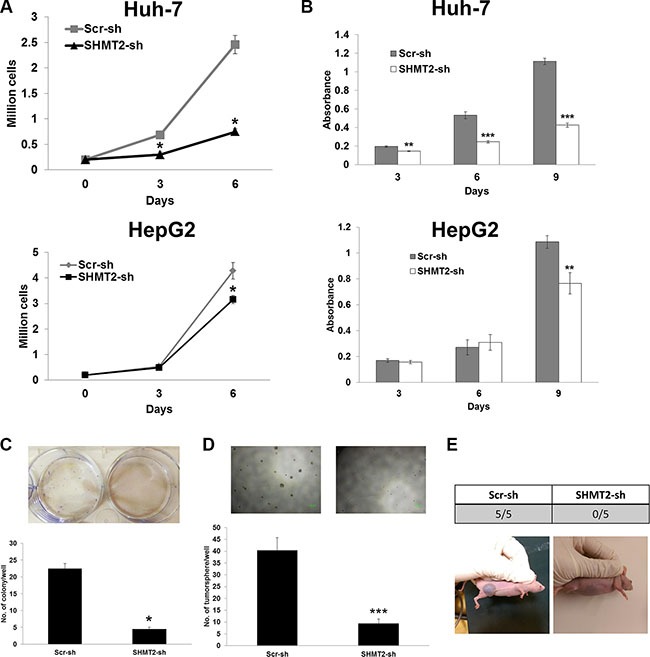
SHMT2 knockdown is able to reduce cell growth and tumorigenicity (**A**) effect of SHMT2 knockdown on Huh-7 and HepG2 cell growth. 2 × 10^5^ cells were seeded into 10 cm culture dish and incubated for 3 and 6 days followed by cell count. The data represent mean ± SEM of three different experiments. *p < 0.05. (**B**) MTT assay to show the effect of SHMT2 knockdown on Huh-7 and HepG2 cell proliferation. 500 cells were seeded into 96-well microplate and incubated for 3, 6 and 9 days. The data represent mean ± SEM of three independent experiments. **p < 0.01, ***p < 0.001. (**C**) effect of SHMT2 knockdown on colony formation in Huh-7 cells. 1000 cells were seeded into 6-well microplate and incubated for 2 weeks followed by crystal violet staining. The data represent mean ± SEM of three different experiments. *p < 0.05. (**D**) effect of SHMT2 knockdown on tumorsphere formation in Huh-7 cells. 200 cells were seeded into ultra-low attachment 96-well microplate and incubated for a week. The data represent mean ± SD of 5 wells. ***p < 0.001. (**E**) effect of SHMT2 knockdown on *in vivo* tumor formation in Huh-7 cells. 5 × 10^6^ cells were subcutaneously inoculated into the right flank of nude mice (*n* = 5). Tumor formation was observed for 7 weeks.

Next, we determined the consequences of SHMT2-knockdown in terms of cell growth and tumorigenicity. We found that SHMT2-knockdown significantly suppressed Huh-7 and HepG2 cell proliferation (Figure 3A–3B). Numbers of colony (Figure 3C) and tumorsphere (Figure 3D) were significantly reduced after SHMT2-knockdown in Huh-7 cells indicating reduced tumorigenicity. Reduced number of tumorsphere was also observed in SHMT2-knockdown HepG2 cells (Supplementary Figure 1C). Following these *in vitro* outcomes, we then examined the *in vivo* tumorigenicity of SHMT2-knockdown Huh-7 cells by inoculating these cells into nude mice. Seven weeks after cell inoculation, no tumor was detected in all five mice inoculated with SHMT2-knockdown cells (Figure 3E). In contrast all five mice inoculated with control cells developed tumors. These findings suggest the importance of SHMT2 in liver cancer cell proliferation and tumorigenesis.

### SHMT2 overexpression increases THLE2 cell proliferation but does not induce malignancy transformation

**Figure 4:**
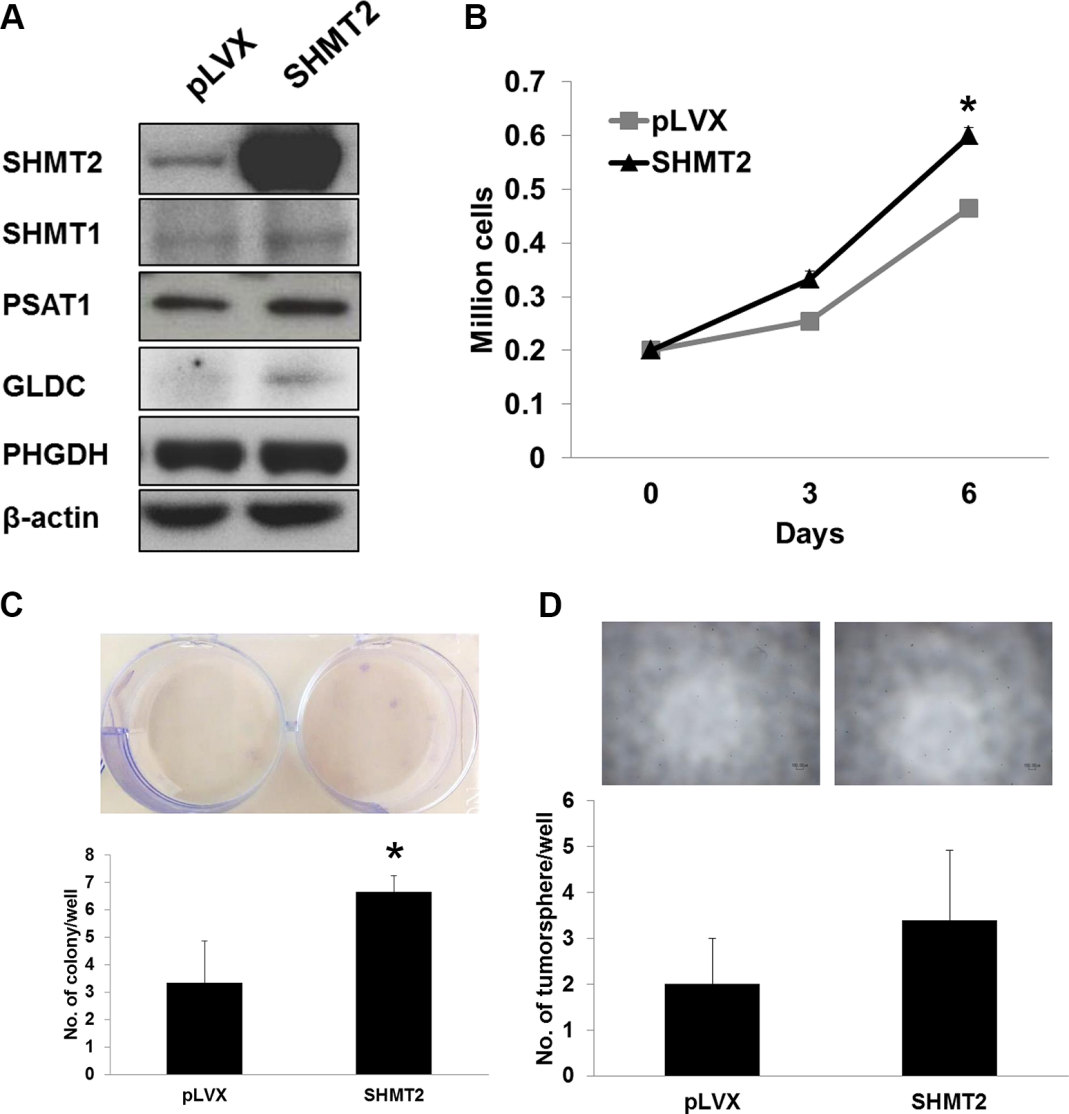
SHMT2 overexpression is insufficient to transform THLE2 normal liver cells to malignancy (**A**) the protein expression of serine-glycine metabolic genes in THLE2 cells expressing SHMT2 vector (SHMT2) versus THLE2 cells expressing empty vector (pLVX). The data are the best representative of three independent experiments. (**B**) effect of SHMT2 overexpression on THLE2 cell growth. 2 × 10^5^ cells were seeded into 10 cm culture dish and incubated for 3 and 6 days followed by cell count. The data represent mean ± SEM of three different experiments. *p < 0.05. (**C**) effect of SHMT2 overexpression on colony formation in THLE2 cells. 1000 cells were seeded into 6-well microplate and incubated for 2 weeks followed by crystal violet staining. The data represent mean ± SEM of three different experiments. *p < 0.05. (**D**) effect of SHMT2 overexpression on tumorsphere formation in THLE2 cells. 200 cells were seeded into ultra-low attachment 96-well microplate and incubated for a week. The data represent mean ± SD of 5 wells.

To assess whether SHMT2 promotes cellular transformation and tumorigenesis, we overexpressed the gene in THLE2 immortalized hepatic cells, as confirmed by quantitative RT-PCR (Supplementary Figure 2A) and Western blot (Figure 4A). We observed an upregulation in GLDC expression while no change in other metabolic genes along the serine-glycine biosynthetic pathway (Supplementary Figure 2A; Figure 4A). However we are not sure whether this upregulation is to metabolize increased amount of glycine of which its accumulation was reported to cause cytotoxicity [18]. The relationship between SHMT1 and SHMT2 appeared to be independent to each other. SHMT2 overexpression was found to promote THLE2 cell growth as measured by cell proliferation (Figure 4B) and MTT assays (Supplementary Figure 2B). The doubling time was reduced from ~112.4 h to ~89.7 h. Even though SHMT2 overexpression enhanced colony formation in THLE2 cells (Figure 4C), the actual colony quantity was still negligible compared to Huh-7 and HepG2 cells. We also found that the number of tumorsphere in THLE2 cells overexpressing SHMT2 was low and not significantly different from the control cells (Figure 4D). Collectively, our results suggest that SHMT2 overexpression is insufficient to promote malignant transformation.

### Huh-7 cells demonstrate maximal SHMT2 activity

**Figure 5:**
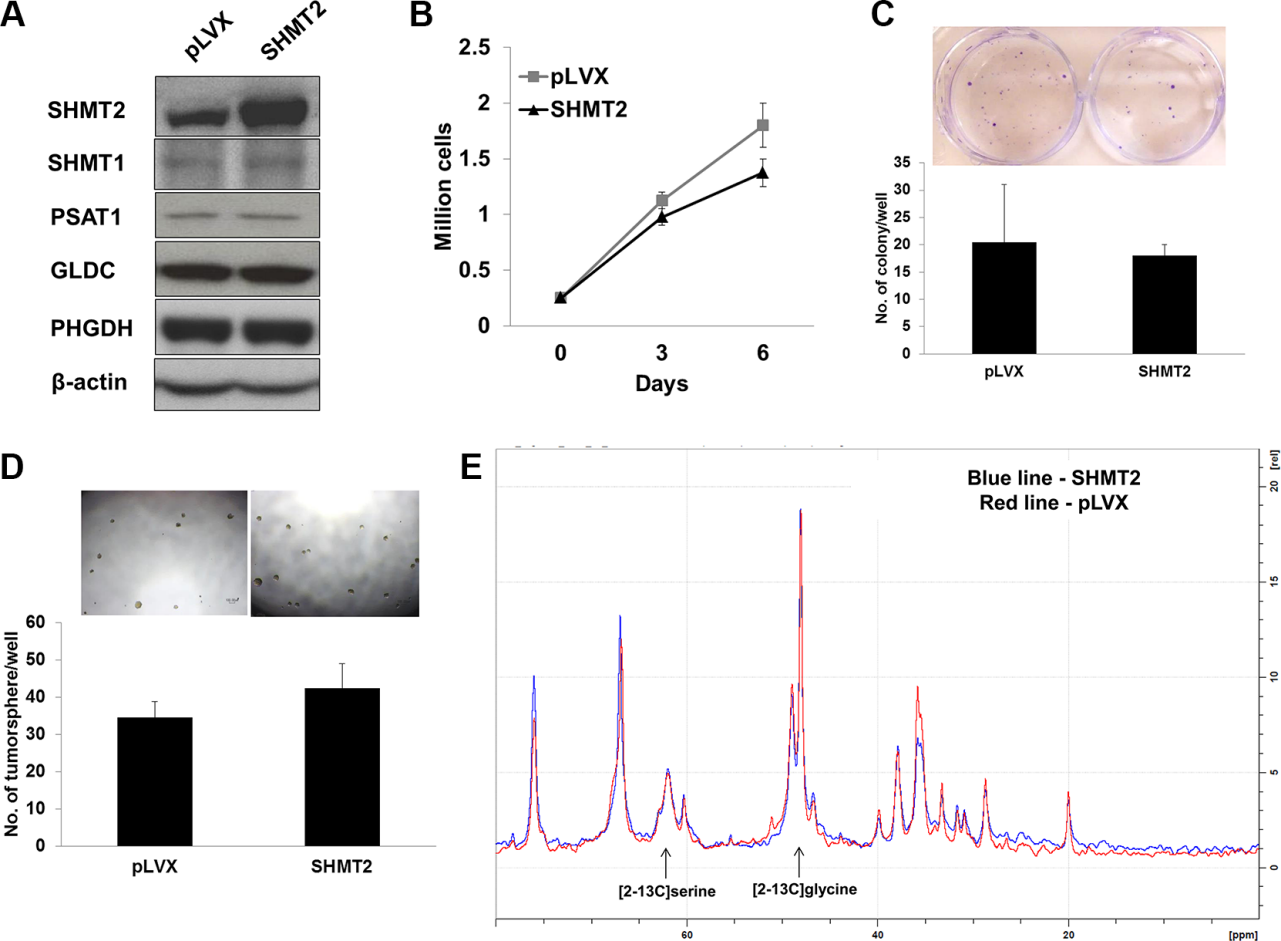
Huh-7 cells exhibit maximal SHMT2 activity (**A**) the protein expression of serine-glycine metabolic genes in Huh-7 cells expressing SHMT2 vector (SHMT2) versus Huh-7 cells expressing empty vector (pLVX). The data are the best representative of three independent experiments. (**B**) effect of SHMT2 overexpression on Huh-7 cell growth. 2 × 10^5^ cells were seeded into 10 cm culture dish and incubated for 3 days and 6 days followed by cell count. The data represent mean ± SEM of three different experiments. (**C**) effect of SHMT2 overexpression on colony formation in Huh-7 cells. 1000 cells were seeded into 6-well microplate and incubated for 2 weeks followed by crystal violet staining. The data represent mean ± SEM of three different experiments. (**D**) effect of SHMT2 overexpression on tumorsphere formation in Huh-7 cells. 200 cells were seeded into ultra-low attachment 96-well microplate and incubated for a week. The data represent mean ± SD of 5 wells. (**E**) NMR spectrum of SHMT2-overexpressed Huh-7 cells (SHMT2) versus control Huh-7 cells (pLVX).

SHMT2 protein is naturally abundant in Huh-7 cells and we further overexpressed this gene to a 3-fold higher level as shown by the mRNA (Supplementary Figure 3A) and protein expressions (Figure 5A). We observed that SHMT2 overexpression did not affect the expression of other metabolic genes along the serine-glycine biosynthetic pathway. SHMT2 overexpression also did not alter Huh-7 cell growth as measured by cell proliferation (Figure 5B) and MTT assays (Supplementary Figure 3B). No significant difference was detected in colony formation (Figure 5C) and tumorsphere population (Figure 5D) in SHMT2-overexpressed Huh-7 cells versus the control cells. To understand these observations, SHMT2 activity was measured *in vitro* by incubating with the ^13^C isotopomer tracer [2–13C] glycine. We found that the product [2–13C] serine concentration was similar between control and SHMT2-overexpressed cells (Figure 5E), suggesting that no difference in SHMT2 activity. Together, these results suggest that SHMT2 catalytic flux is saturated in Huh-7 cells whereby further expression was redundant. The need for full activity of SHMT2 in cancer cells also implies that it is a crucial gene in tumorigenicity, making it an important target.

### Inhibiting SHMT2 reduces tumor incidence and tumor growth

**Figure 6:**
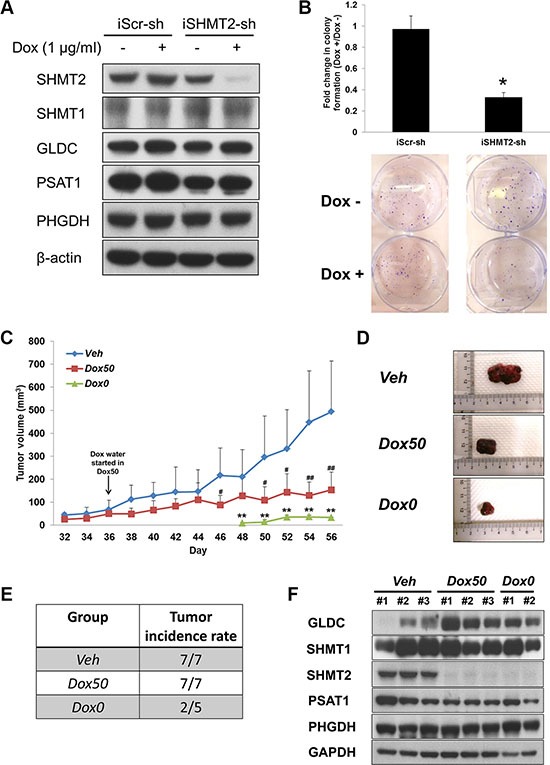
SHMT2 inhibition is able to reduce tumor growth as well as tumor incidence (**A**) the protein expression of serine-glycine metabolic genes in Huh-7 cells expressing inducible SHMT2 shRNA (iSHMT2-sh) versus Huh-7 cells expressing inducible scramble shRNA (iScr-sh). The cells were treated with 4 days of doxycycline hyclate (1 μg/ml) before harvested for Western blot. The data are the best representative of three independent experiments. (**B**) effect of inducible SHMT2 knockdown on colony formation in Huh-7 cells. 1000 cells were seeded into 6-well microplate and incubated for 2 weeks followed by crystal violet staining. The data represent fold change in the number of colony with or without treatment of 1 μg/ml doxycycline hyclate (Dox +/Dox –). *p < 0.05. (**C**) tumor volume progression of all treatment groups. 5 × 10^6^ Huh-7-iSHMT2sh cells were subcutaneously injected into the right flank of nude mice. Data represent mean tumor volume ± SD. Veh vs Dox50: ^#^*p* < 0.05, ^##^*p* < 0.01. Veh vs Dox0: **p < 0.01. (**D**) representative pictures of extracted tumors from each treatment group. (**E**) tumor incidence rate in each treatment group. (**F**) the protein expression of serine-glycine metabolic genes in tumor tissues from each treatment group. The data are the best representative of three independent experiments.

To explore the therapeutic potential of inhibiting SHMT2, a tetracycline-inducible SHMT2-knockdown Huh-7 cell line (iSHMT2-sh) was created. After incubation with doxycycline (Dox) for 4 days, SHMT2 gene expression was shown to be successfully suppressed in these cells versus no change in the control cells (Figure 6A and Supplementary Figure 4A). Similarly, Dox-induced SHMT2 inhibition caused decreases in cell growth (Supplementary Figure 4B), colony formation (Figure 6B) and tumorsphere population (Supplementary Figure 4C). No significant change was observed in SHMT1, GLDC, PSAT1 and PHGDH gene and protein expressions after Dox treatment.

To validate the *in vitro* results in an animal model, we tested the efficacy of SHMT2 inhibition in arresting tumor development by implanting these cells subcutaneously in nude mice. A total of three groups were formed and they were supplied with the respective treatments: *Veh* − 2 % sucrose water only; *Dox50* **-** Dox water (2% sucrose + 2 mg/ml doxycycline hyclate) after tumor size reaches 50 mm^3^; *Dox0* **-** Dox water immediately after cell inoculation. It was observed that tumor growth in mice administered with Dox in their drinking water immediately after cell implantation was severely restricted (Figure 6C), coupled with a delayed tumor onset. Only 2 out of 5 mice in group *Dox0* developed tumors, whereas all mice in groups *Veh* and *Dox50* developed tumors (Figure 6E). When Dox treatment began in group *Dox50* on Day 36, tumor growth was impeded and xenograft sizes were significantly smaller after 10 days (Figure 6C–6D).

SHMT2 protein expression in excised tumors clearly showed the inhibition of the enzyme in both Dox-treated groups, *Dox50* and *Dox0* (Figure 6F). Interestingly GLDC expression was found to be upregulated after Dox treatment and this observation was similar to our *in vitro* data with stable SHMT2 knockdown (Figure 2A–2B). No significant change was observed in SHMT1, PSAT1 and PHGDH protein expression for all three groups.

### Tumorigenicity of SHMT2 is mediated via glycine

**Figure 7:**
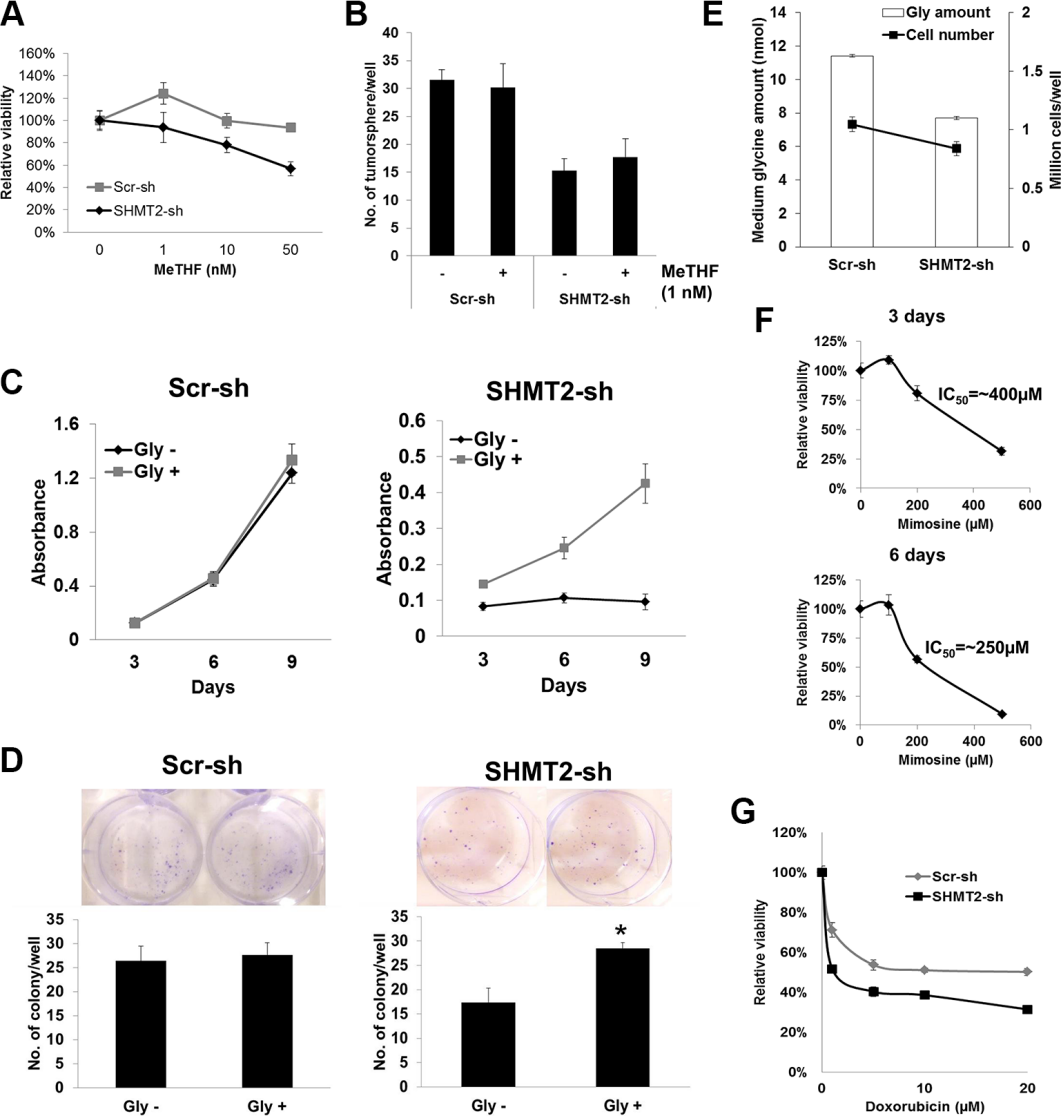
The tumorigenicity of SHMT2 is mediated via glycine rather than MeTHF (**A**) MTT assay to show the effect of MeTHF on the cell proliferation of SHMT2-knockdown Huh-7 cells (SHMT2-sh) versus control Huh-7 cells (Scr-sh). 500 cells were seeded into 96-well microplate and incubated for 8 days with increasing concentrations of MeTHF. The absorbance was normalized to the PBS control. The data represent mean ± SEM of three independent experiments. (**B**) effect of MeTHF on tumorsphere formation in SHMT2-knockdown Huh-7 cells versus control Huh-7 cells. 200 cells were seeded into ultra-low attachment 96-well microplate and incubated for a week with or without 1 nM MeTHF. The data represent mean ± SD of 5 wells. (**C**) MTT assay to show the effect of exogenous glycine to the cell proliferation of SHMT2-knockdown Huh-7 cells versus control Huh-7 cells. 500 cells were seeded into 96-well microplate and incubated for 3, 6 and 9 days with/without added glycine. The data represent mean ± SEM of three independent experiments. (**D**) effect of exogenous glycine on colony formation in SHMT2-knockdown Huh-7 cells versus control Huh-7 cells. 1000 cells were seeded into 6-well microplate and incubated for 2 weeks, with/without added glycine, followed by crystal violet staining. The data represent mean ± SEM of three different experiments. *p < 0.05. (**E**) effect of SHMT2 knockdown on glycine uptake in Huh-7 cells. 3 × 10^5^cells were seeded into 10 cm culture dish followed by collection of medium for glycine assay after 4-day incubation. (**F**) effect of L-mimosine on Huh-7 cell proliferation. 500 cells were seeded into 96-well microplate followed by L-mimosine treatment for 3 days (top panel) and 6 days (bottom panel). The data represent mean ± SEM of three independent experiments. (**G**) MTT assay to show the effect of doxorubicin to the viability of SHMT2-knockdown Huh-7 cells versus control Huh-7 cells. 10^4^ cells were seeded into 96-well microplate followed by doxorubicin treatment for 48 h. The absorbance was normalized to the vehicle control. The data represent mean ± SEM of three independent experiments.

There are two sources of MeTHF in the serine-glycine pathway, via SHMT2- and GLDC-mediated reactions respectively. To determine whether the decreased production of this folate metabolite is accountable for the reduced tumorigenicity in SHMT2- or GLDC-knockdown cells, its supplementation was introduced to the cell culture. MeTHF-supplemented cells exhibited slower growth from 1 nM MeTHF onwards (Figure 7A and Supplementary Figure 5A). Tumorsphere formation in MeTHF-supplemented Huh-7 cells with/without SHMT2-knockdown remained unchanged at 1 nM MeTHF (Figure 7B). Similarly, colony formation was not altered in MeTHF-supplemented Huh-7 cells with/without GLDC-knockdown (Supplementary Figure 5B). On the other hand, when exogenous glycine was supplemented to SHMT2-knockdown cells incubated with glycine-deficient medium, both cell growth (Figure 7C) and colony formation (Figure 7D) were improved. Moreover, we found that SHMT2-knockdown Huh-7 cells exhibited increased glycine uptake as shown by the significantly lower glycine amount (< 70%) in the medium as compared to the control Huh-7 cells (Figure 7E). These data suggest that SHMT2-knockdown cells tried to compensate the loss of SHMT2 function by increasing glycine uptake. Together, these results suggest that the tumorigenicity of SHMT2 is possibly mediated via glycine rather than MeTHF.

### SHMT2 inhibition increases the chemosensitivity of Huh-7 cells

There is no potent SHMT2 inhibitor currently available. As of now only a natural plant amino acid L-mimosine has been reported to inhibit SHMT [19], by disrupting deoxynucleotide metabolism and thus DNA synthesis [20]. Our data showed that L-mimosine treatment inhibited SHMT2 protein expression but not SHMT1 in Huh-7 cells (Supplementary Figure 5D). However, this compound is also able to suppress PSAT1 suggesting that its effect is not specific. Though L-mimosine was not particularly potent, it did however suppress Huh-7 cell viability with an IC_50_ of about 400 μM and 250 μM after 3- and 6-day incubation, respectively (Figure 7F). In addition, SHMT2 knockdown was found to increase the chemosensitivity of Huh-7 cells to the cytotoxicity of doxorubicin, a chemotherapeutic agent (Figure 7G). This finding suggests the possibility of combined therapy of SHMT2 inhibitor and chemotherapeutic agent which might achieve higher efficacy in cancer treatment.

## DISCUSSION

Recent studies have highlighted the important role of serine and glycine in supporting tumorigenesis [4, 11, 12]. To fuel the increased demand of *de novo* serine/glycine biosynthesis and one-carbon metabolism, metabolic genes along the pathway including PHGDH [8], SHMT2 [11] and GLDC [16] are ubiquitously upregulated in cancer. While SHMT1 is expressed to different degrees across carcinomas [21, 22], there is not yet any report concerning the role of SHMT2 in human HCC. The goal in this study is therefore to identify the potential of SHMT2 as a therapeutic target in HCC, given its critical role in serine-glycine inter-conversion [23]. We found that SHMT2 is upregulated in human HCC including Huh-7, HepG2 and Hep3B cell lines. Knocking down SHMT2 in Huh-7 and HepG2 cells resulted in a reduction in cell proliferation and tumorigenicity demonstrating a therapeutic effect. It was observed that overexpressing SHMT2 neither altered the tumorigenicity of Huh-7 cells, nor SHMT2 activity as measured by C13 NMR. The “saturated” activity of SHMT2 in Huh-7 cells somewhat suggests its critical role in tumorigenesis. This phenomenon contrasts with that seen in the upstream metabolic enzyme PSAT1, in which overexpression of the protein stimulates cell growth, increases tumorigenicity and promotes chemo-resistance in SW480 colon carcinoma cells [24]. Therefore, the metabolic genes along the serine-glycine biosynthetic pathway appear to exhibit different oncogenic behaviors across cancer phenotypes.

We found that SHMT2 overexpression increased THLE2 cell growth but did not alter tumorigenicity. This suggests that SHMT2 alone does not transform hepatocytes to malignancy. Since SHMT2 overexpression increases THLE2 cell growth, the possibility of overexpressing this gene in regenerating tissues remains to be explored. On the other hand, SHMT2-overexpressed 3T3 mouse embryonic fibroblast cells did indeed generate tumor xenograft after inoculation into nude mice, albeit at a low incidence rate (1 out of 6 mice) [16]. Therefore the oncogenic characteristic of SHMT2 appears to depend on cell lineages. Interestingly, we also found that GLDC is upregulated in SHMT2-overexpressed THLE2 cells, whereas the similar upregulation was also observed in SHMT2-overexpressed 3T3 cells [16]. Whether GLDC upregulation is responsible for the accelerated cell proliferation in SHMT2-overexpressed cells remains to be explored.

We also studied the *in vivo* tumorigenic potential of SHMT2-knockdown Huh-7 cells by inoculating these cells into nude mice. No tumor was formed in these mice, in contrast with those inoculated with control Huh-7 cells. A study by Ye *et al*. on the other hand showed that nude mice inoculated with SHMT2-knockdown Kelly neuroblastoma cells did indeed develop tumors, albeit at a slower growth rate compared to its control group [11]. Again this indicates the importance of understanding the heterogeneity of SHMT2 across cancer phenotypes. We used tetracycline-inducible SHMT2-knockdown tumor xenograft mouse model to inhibit SHMT2 expression at certain tumor sizes in order to mimic clinical situation. The therapeutic effect of inhibiting SHMT2 is particularly evident in the *DOX50* group, in which tumor size was 70% lower than that in the vehicle group after 20 days of treatment. Furthermore, the reduced tumor incidence and delayed tumor onset in *DOX0* group illustrated the preventive advantage that a subdued serine-glycine metabolic flux could bring about. Even though increased GLDC gene expression was observed in mice administered with Dox water, tumor growth was not rescued. This suggests that the HCC cells may have attempted metabolically to compensate for the reduction in *de novo* biosynthesis of glycine via GLDC upregulation, in a bid to maintain MeTHF supply for cell proliferation. Therefore we next investigated the importance of the downstream products of SHMT2 catalysis, glycine and MeTHF.

In this study, it was observed that *in vitro* supplementation of MeTHF could not reverse the impairments in cell growth and tumorigenicity as a result of SHMT2 or GLDC knockdown. These results suggest that MeTHF alone is not a major contributing factor to tumorigenesis. Interestingly, a higher dose of MeTHF supplementation was found to inhibit rather than promote cell proliferation. Whether excess MeTHF would increase the conversion of glycine to serine, resulting in decreased level of glycine that causes inhibition in cell proliferation, remains to be investigated. Glycine on the other hand may play a major role in tumorigenesis [12, 16]. Two-thirds of intracellular glycine supply is synthesized endogenously [12]. We observed that providing exogenous glycine improved both cell growth and tumorigenicity of SHMT2-knockdown cells. Therefore, it appeared that the cells increased exogenous glycine utilization when *de novo* glycine biosynthesis is suppressed. Indeed, we found that the medium glycine amount was lowered in SHMT2-knockdown cells indicating increased glycine uptake. These results also suggest that glycine is a more dominant factor in HCC oncogenesis than MeTHF. Previous studies of metabolic tracing with [13C] glycine demonstrated that glycine is utilized in *de novo* purine biosynthesis either through direct incorporation into purine backbone or via glycine cleavage system to yield one-carbon units [12], highlighting its importance in nucleotide production. Paradoxically, accumulation of glycine causes cytotoxicity, which can be attributed to its conversion into toxic metabolites such as methylglyoxal and aminoacetone by GCAT [18]. Therefore we may argue that tumor cells with high SHMT2 level, such as Huh-7 cells, are sensitive to GLDC inhibition because excess glycine is not metabolized rapid enough via the glycine cleavage system, as evident from our results seen in the Huh-7 cells with GLDC knockdown (Supplementary Figure 5C).

Our *in vitro* and *in vivo* data suggest that SHMT2 is one of the building blocks, rather than a key driver, in liver tumorigenesis. Indeed, SHMT1 and SHMT2 are direct targets of oncogene Myc, thereby illustrating the intrinsic link between tumorigenesis and cellular metabolism [10]. Moreover, genomic instability caused by nucleotide deficiency in early oncogenesis might be overcome by SHMT2 upregulation to sustain tumor progression [25]. In addition, Kim *et al*. established a responsive relationship between glycolysis and serine-glycine pathway that conditions glioblastoma cells to survive in a hypoxic microenvironment, primarily via the modulation of carbon flux by pyruvate kinase M2 (PKM2) and SHMT2 [18, 26]. Clinically breast cancer patients with high expression of SHMT2 showed poorer survival outcome [27]. Taken together, we present substantial evidence corroborating SHMT2 as a potential metabolic target in HCC and believe that efforts to discover its inhibitors for clinical therapy should be doubled. Since SHMT2 is also required in normal cells, thus there is a possibility that its inhibitor may cause toxicity in highly-replicating non-transformed cells such as human mammary epithelial cells and human bronchial epithelial cells [12].

## MATERIALS AND METHODS

### Cell culture

THLE2, Hep3B, and HepG2 cell lines were purchased from ATCC. HEK293T and Huh-7 cell lines were generous donations from Dr. Weiping Han in the institute. HEK293T, Hep3B, HepG2 and Huh-7 cells were cultured in DMEM supplemented with 10% FBS and 1% penicillin-streptomycin. THLE2 cells were cultured in LHC-8 medium (Life Technologies) supplemented with 5 ng/ml EGF, 70 ng/ml phosphoethanolamine, 9.2 ng/ml retinoid acid, 10% FBS and 1% penicillin-streptomycin. All cell cultures were maintained at 37°C in a humidified incubator with 5% CO_2_.

### Reverse transcription and quantitative real-time PCR

Cells or tissues were mixed with TRIzol reagent (Invitrogen). Total RNA was extracted according to the manufacturer's protocol. 2 μg total RNA was treated with DNase (Invitrogen) followed by reverse transcription (Thermo Scientific). Quantitative real-time PCR was performed with StepOnePlus™ System (Applied Biosystems) using Maxima SYBR Green qPCR Master Mix (Thermo Scientific). The expression of mRNA was calculated using 2^-ΔΔCt^ method. The sequences of PCR primers (Integrated DNA Technologies) were listed in Supplementary Table 1.

### Western blot

Cell or tissue lysate was mixed with Laemmli sample buffer before resolved in 10 or 12% SDS/PAGE gel, which in turn electroblotted onto a nitrocellulose membrane using iBlot^®^ (Thermo Fisher Scientific). The membrane was blocked in 5% non-fat milk (Bio-Rad) followed by overnight incubation with antibody of interest (dilution 1:1000) at 4°C. After PBST wash, the membrane was incubated with the respective secondary antibody (dilution 1:10000) for an hour at room temperature. After addition of ECL plus (GE Healthcare) to the membrane, the chemiluminescence was exposed to an X-ray film (GE Healthcare). The sources of each antibody were listed in Supplementary Table 2.

### Tumorsphere assay

This assay was performed as described previously [28]. Briefly, the suspension of 200 cells in 200 μl tumorsphere medium (DMEM/F12 medium supplemented with 1× B27 (Invitrogen), 20 ng/ml EGF, 10 ng/ml bFGF, 5 μg/ml insulin and 0.4% bovine serum albumin) was added into 96-well ultra-low attachment microplate. The plate was sealed with laboratory tape to avoid evaporation. After a week of incubation in CO_2_ chamber, the amount of tumorsphere was measured using a phase-contrast microscope (TS-100F, Nikon).

### Colony forming assay

This assay was done as described previously [29]. Briefly, 1000 cells were seeded into 6-well microplate and fresh medium was replenished every 3 days. At the end of incubation, the medium was removed followed by a PBS wash. The cells were then stained with 0.5% crystal violet solution (with 6% glutaraldehyde, Sigma-Aldrich) for 20 mins. After removing the crystal violet solution, the plate was carefully rinsed with water to remove excessive stain. The plate was left to air-dry at room temperature followed by colony count.

### Cell proliferation assay

10 ml of 0.2 or 0.3 million cells were seeded into 10 cm culture dish and cultured for 3 and 6 days. Fresh medium was replenished every 3 days. After trypsinization, cell count was done via trypan blue exclusion with a haemocytometer.

### MTT assay

After cell count, 500 or 1000 cells in 100 μl medium were added into 96-well microplate. For certain experiments, the cells were either treated with MeTHF (Adooq Bioscience) or L-mimosine (Sigma-Aldrich). At the end of incubation, 10 μl MTT solution (5 mg/ml, Sigma-Aldrich) was added into each well for 3 h. The medium was carefully removed by pipetting without disturbing the formazan crystals at the bottom. 100 μl DMSO was added to dissolve the crystals followed by absorbance measurement (Infinite^®^ M200, Tecan) at 570 nm.

### Glycine assay

This assay was performed according to the manufacturer's protocol. Briefly, 3 × 10^5^ cells were seeded into 10 cm culture dish in medium supplemented with 100 μM glycine. After 4-day incubation, the cell medium was collected followed by filtration to remove cells. The sample was deproteinized using 10 kDa spin column. 50 μl of the sample was loaded into 96-well black/clear bottom microplate followed by 50 μl reaction mix. The plate was incubated under dark condition for 1 h in room temperature. At the end of incubation, the fluorescence (Ex/Em = 535/587 nm) was measured using a microplate reader (SpectraMax M2, Molecular Devices).

### Lentiviral packaging and infection

The sequences of each construct were listed in Supplementary Table 3. All constructs were verified with DNA sequencing before used in the experiments. SHMT2 shRNA was synthesized according to the sequence as described previously [12]. pLKO vector was used for gene knockdown study while pLVX vector was used for gene overexpression study. HEK293T cells were seeded into 10 cm culture dish for overnight incubation. These cells were transfected with plasmids of interest using FuGENE transfection reagent (Promega). The cell medium containing virus particles was collected at 48 h and 72 h post-transfection. After filtration (0.45 μm filter) and addition of polybrene (8 μg/ml), the cell medium was added to the cells of interest (Huh-7, HepG2 or THLE2 cells) for viral infection. Fresh medium was replenished at the next day. Antibiotic selection (2.0 μg/ml puromycin) was started at 48 h post-infection.

### Tumor xenograft mouse model

All animal procedures were approved by Institutional Animal Care and Use Committee from A*STAR Singapore. 5 million cells were resuspended at 1:1 ratio of matrigel (BD Biosciences) and serum-free medium before inoculated subcutaneously to the right flank region of nude mice. For stable SHMT2-knockdown xenograft mouse model, 2 groups of mice were formed (*n* = 5): control Huh-7 cells (Scr-sh) and SHMT2-knockdown Huh-7 cells (SHMT2-sh). Tumor formation was examined for 7 weeks post cell inoculation. For tetracycline-inducible SHMT2-knockdown tumor xenograft mouse model, 3 groups of mice were formed and they were inoculated with tetracycline-inducible SHMT2-knockdown Huh-7 cells. Group *Veh* (*n* = 7) was given 2% sucrose water after cell inoculation. Group *Dox50* (*n* = 7) was given 2% sucrose water after cell inoculation, and when tumor volume reached 50 mm^3^, the mice were given Dox water (2 mg/ml doxycycline hyclate + 2% sucrose). Group *Dox0* (*n* = 5) was given Dox water immediately after cell inoculation. Tumor size was measured with a caliper followed by calculation of tumor volume using the formula: volume = (width^2^ × length)/2. At the end of the study, tumor xenografts were harvested, snap frozen, and stored at −80°C.

### Nuclear magnetic resonance (NMR)

Cells were incubated in glycine-deficient medium (Sigma-Aldrich) supplemented with 10 mM [2–13C] glycine for 6 days. Fresh medium was replenished every 3 days. 50 million cells were subsequently lysed with magnetic beads followed by centrifugation at 14000 rpm. The supernatant was put into a 5-mm NMR sample tube for analysis (Bruker 400 MHz NMR Spectrometer). 3300 scans were acquired over a sweep width of 25252 Hz.

### Statistical analysis

The statistical analyses for group comparisons were performed using unpaired Student's *t*-test and one-way analysis of variance (ANOVA). Significance was represented with asterisk as follow: *p < 0.05, **p < 0.01, ***p < 0.001.

## SUPPLEMENTARY MATERIALS FIGURES AND TABLES


