# Supplementary material for: Downregulating serine hydroxymethyltransferase 2 (SHMT2) suppresses tumorigenesis in human hepatocellular carcinoma
Source: Oncotarget. 2016 Jul 6;7(33):53005–17. doi: 10.18632/oncotarget.10415 (PMC5288164; doi:10.18632/oncotarget.10415)
Supplement: Supplementary file 1 [file oncotarget-07-53005-s001.pdf]

Downregulating serine hydroxymethyltransferase 2 (SHMT2) suppresses tumorigenesis in human hepatocellular carcinoma

Supplementary Materials

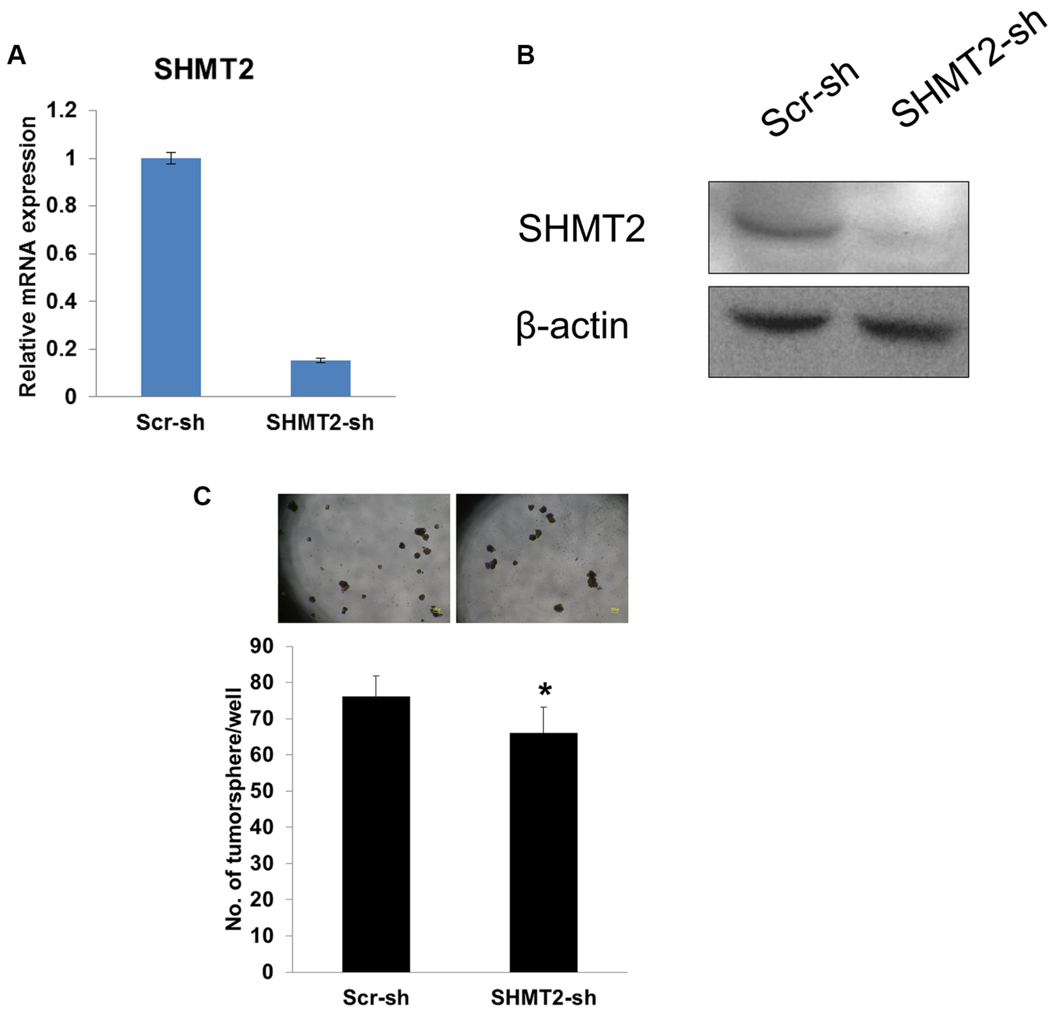

Supplementary Figure S1: (A) The mRNA expression of SHMT2 in HepG2 cells expressing SHMT2 shRNA (SHMT2-sh) in relative to HepG2 cells expressing scramble shRNA (Scr-sh). The data represent mean ± SD of triplicate PCR reactions. (**B**) the protein expression of SHMT2 in SHMT2-knockdown HepG2 cells versus scrambled control HepG2 cells. The data are the best representative of three independent experiments. (**C**) effect of SHMT2 knockdown on tumorsphere formation in HepG2 cells. 200 cells were seeded into ultra-low attachment 96-well microplate and incubated for a week. The data represent mean ± SD of 5 wells. *$p < 0.05$.

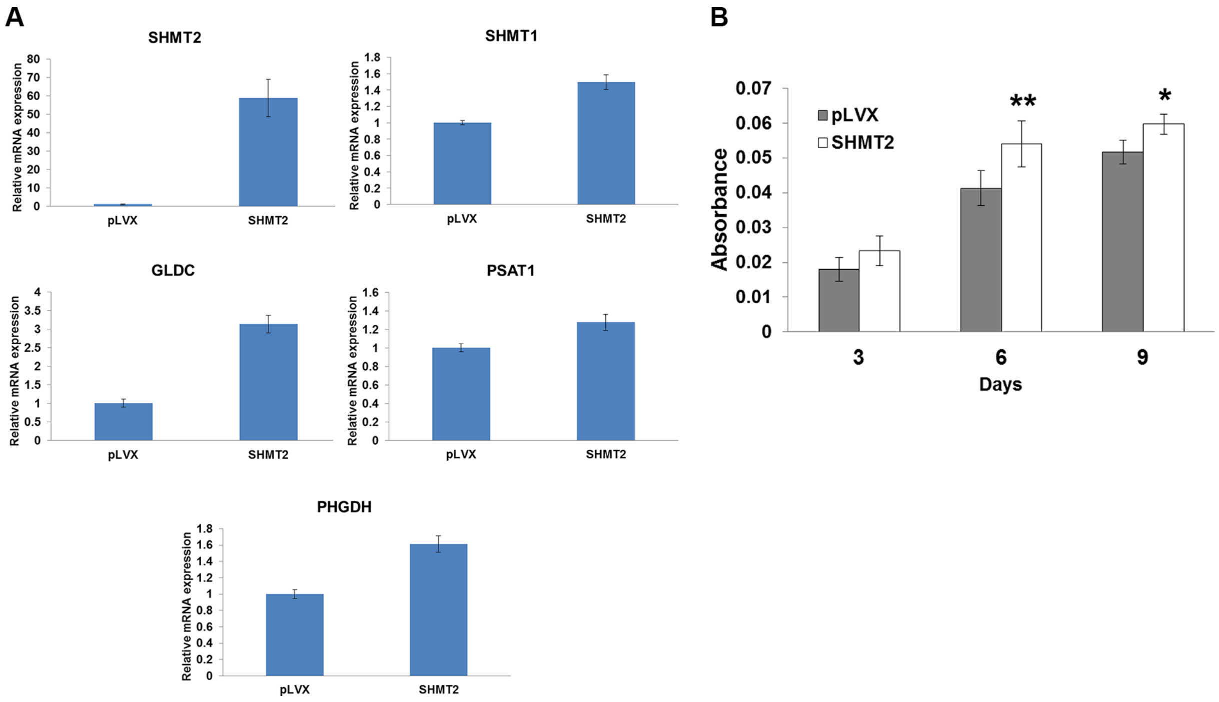

Supplementary Figure S2: (**A**) The mRNA expression of serine-glycine metabolic genes in THLE2 cells expressing SHMT2 vector (SHMT2) in relative to THLE2 cells expressing empty vector (pLVX). The data represent mean ± SD of triplicate PCR reactions. (**B**) MTT assay to show the effect of SHMT2 overexpression to THLE2 cell proliferation. 1000 cells were seeded into 96-well plate and incubated for 3, 6 and 9 days. The data represent mean ± SEM of three different experiments. $^{*}p < 0.05$, $^{**}p < 0.01$.

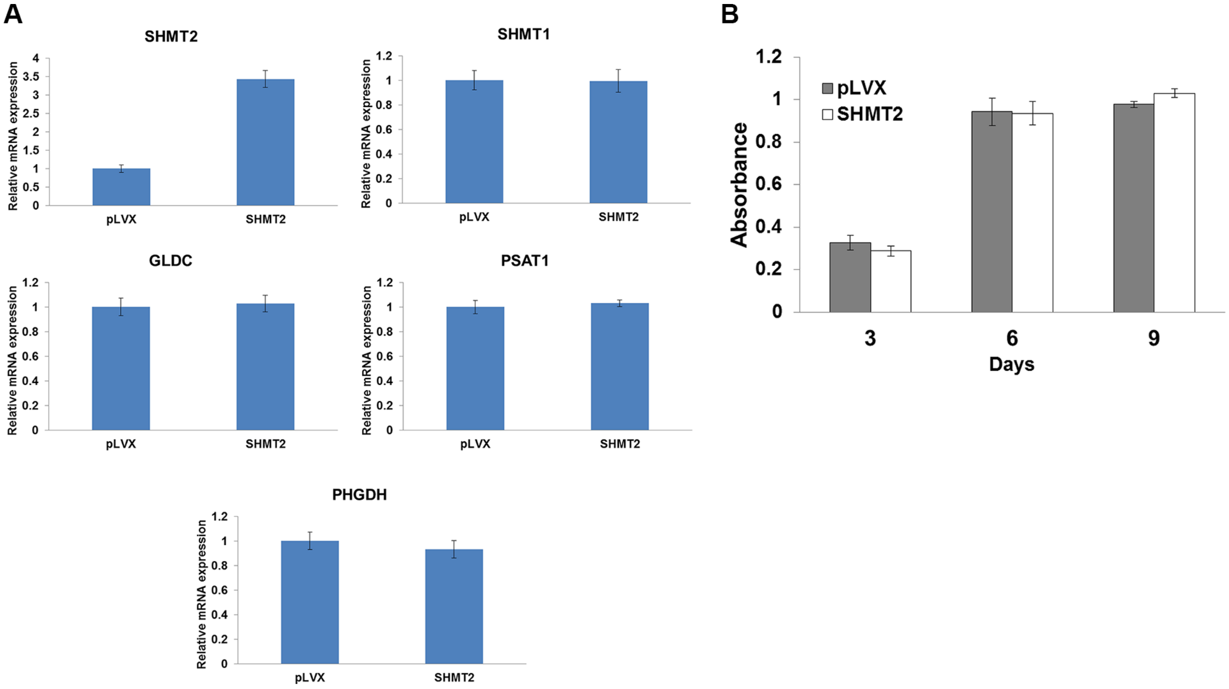

Supplementary Figure S3: (**A**) The mRNA expression of serine-glycine metabolic genes in Huh-7 cells expressing SHMT2 vector (SHMT2) in relative to Huh-7 cells expressing empty vector (pLVX). The data represent mean ± SD of triplicate PCR reactions. (**B**) MTT assay to show the effect of SHMT2 overexpression to Huh-7 cell proliferation. 1000 cells were seeded into 96-well microplate and incubated for 3, 6 and 9 days. The data represent mean ± SEM of three independent experiments.

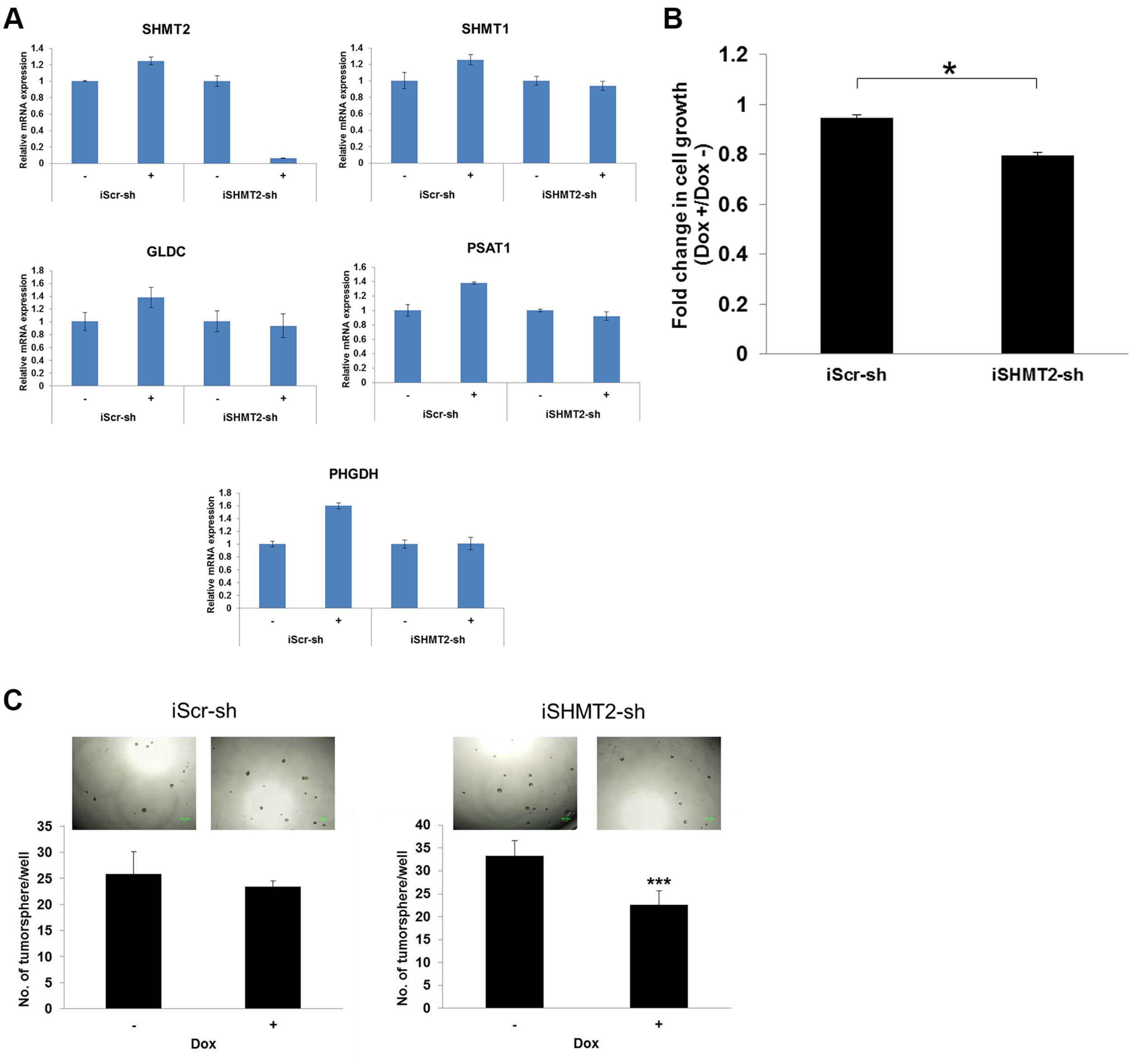

Supplementary Figure S4: (**A**) The mRNA expression of serine-glycine metabolic genes in Huh-7 cells expressing inducible SHMT2 shRNA (iSHMT2-sh) or scramble shRNA (iScr-sh) with/without 4-day treatment of 1 µg/ml doxycycline hyclate. The data represent mean ± SD of triplicate PCR reactions. (**B**) effect of inducible SHMT2-knockdown on Huh-7 cell growth. 3×10^5 cells were seeded into 10 cm culture dish and incubated for 4 days with or without doxycycline hyclate (1 µg/ml). The data represent the ratio of cell growth with/without doxycycline hyclate treatment (Dox +/Dox −). The data represent mean ± SEM of three different experiments. $*p < 0.05$. (**C**) effect of inducible SHMT2-knockdown on tumorsphere formation in Huh-7 cells. 200 cells were seeded into ultra-low attachment 96-well microplate and incubated for a week with/without doxycycline hyclate (1 µg/ml). The data represent mean ± SD of 5 wells. $***p < 0.001$.

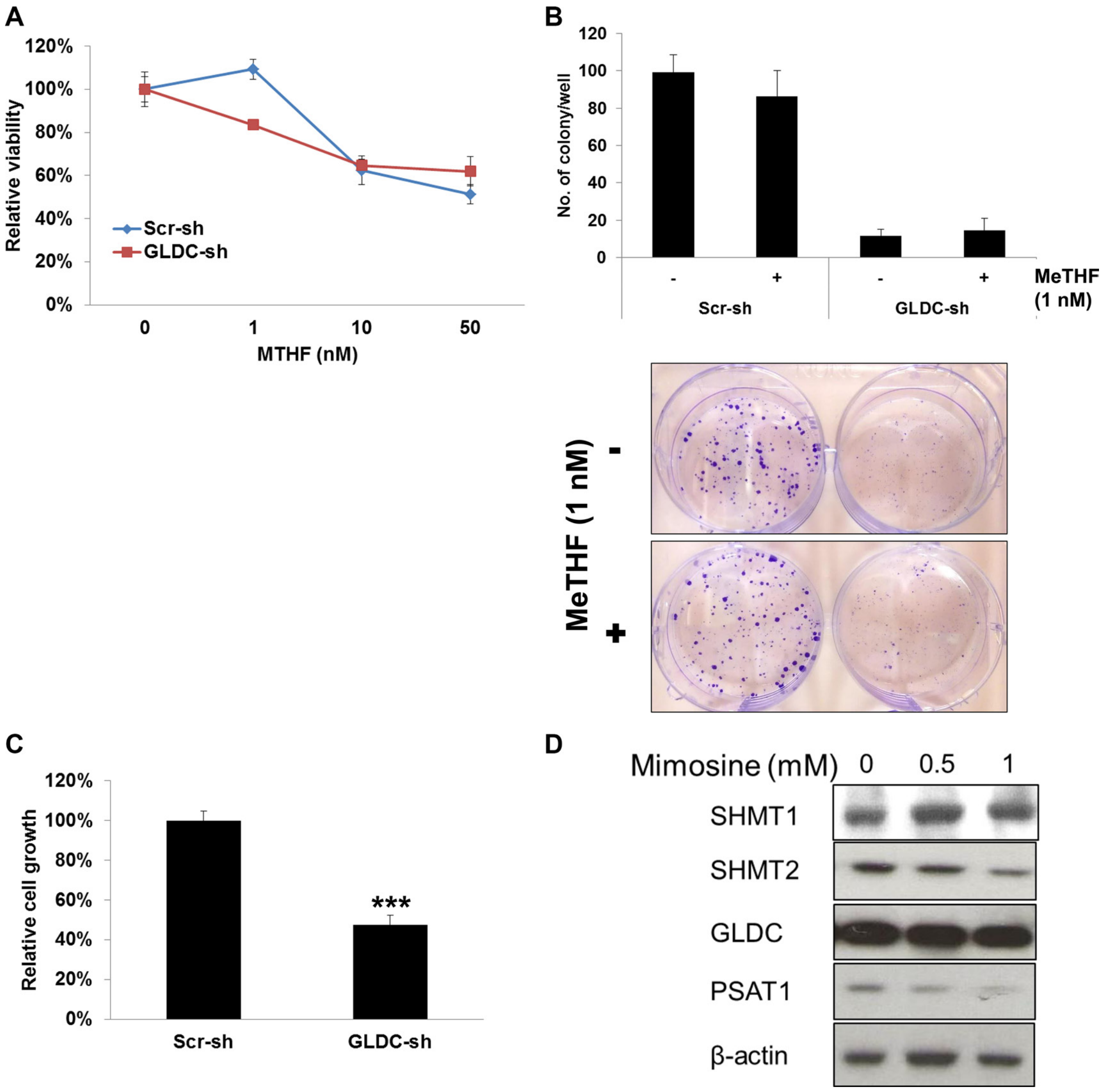

Supplementary Figure S5: (**A**) MTT assay to show the effect of MeTHF on the cell proliferation of Huh-7 cells expressing GLDC shRNA (GLDC-sh) versus Huh-7 cells expressing scramble shRNA (Scr-sh). 500 cells were seeded into 96-well microplate and incubated for 8 days with increasing concentrations of MeTHF. The absorbance was normalized to the PBS control. The data represent mean ± SEM of three independent experiments. (**B**) effect of MeTHF on colony formation in GLDC-knockdown Huh-7 cells. 1000 cells were seeded into 6-well microplate and incubated for 2 weeks with/without 1 nM MeTHF followed by staining with crystal violet solution. (**C**) MTT assay to show the cell growth of Huh-7 cells with GLDC-knockdown. 500 cells were seeded into 96-well microplate and incubated for 6 days. The absorbance was normalized to Scr-sh. The data represent mean ± SEM of three independent experiments. ***$p < 0.001$. (**D**) the protein expression of serine-glycine metabolic genes in Huh-7 cells after 48 h L-mimosine treatment. The data are the best representative of three independent experiments.

Supplementary Table S1: Quantitative RT-PCR primer sequences

Species	Gene	F or R	Sequence
Human	SHMT1	F	TCCTCACATGACAAGATGCTG
		R	ATTCTCCGAGGCAATCAGC
	SHMT2	F	CGAGTTGCGATGCTGTACTT
		R	CTGCGTTGCTGTGCTGAG
	GLDC	F	CCAGACACGACGACTTCGC
		R	CAATTCATCAATGCTCGCCAG
	PSAT1	F	GTTTGCCCTCCGAGAGTG
		R	AATCCACTCCAGAACCAAGC
	PHGDH	F	ATTGCTGTTCAGTTCGTGG
		R	AGAGAAGGCACTGGTAAGG
	TBP	F	ACTCCACTGTATCCCTCCCC
		R	TATATTCGGCGTTTCGGGCA

Supplementary Table S2: List of antibodies

Antibody	Source	Cat. No.
SHMT1	Santa cruz	sc-365203
SHMT2	Santa cruz	sc-25064
PSAT1	Santa cruz	sc-133929
PHGDH	Santa cruz	sc-100317
GLDC	Cell Signaling	12794
β-actin	Cell Signaling	4970
GAPDH	Cell Signaling	2118
chicken anti-goat IgG-HRP	Santa cruz	sc-2953
chicken anti-mouse IgG-HRP	Santa cruz	sc-2954
chicken anti-rabbit IgG-HRP	Santa cruz	sc-2955

Supplementary Table S3: shRNA sequences

System	Gene	Sequence
Stable and Tet-on	SHMT2	CGGAGAGTTGTGGACTTTATA
	Scramble	ACAACAGCCACAACGTCTATA
Stable	GLDC	CCACGGAAACTGCGATATTAAC
	Scramble	ACAACAGCCACAACGTCTATA
